# Nobel committee honors tumor immunologists

**DOI:** 10.1186/s13046-018-0937-6

**Published:** 2018-10-30

**Authors:** Anand Rotte, Gabriella D’Orazi, Madhuri Bhandaru

**Affiliations:** 1 Clinical & Regulatory Affairs, Nevro Corp, Redwood City, CA 94065 USA; 2 Depertment of Medical Sciences, University “G. D’Annunzio”, Chieti, Italy; 3 Department of Research, IRCCS Regina Elena National Cancer Institute, Rome, Italy; 4 Department of Dermatology and Skin Science, University of British Columbia, Vancouver, Canada

**Keywords:** Immunotherapy, Immune checkpoints, CTLA-4, PD-1, PD-L1, Anti-tumor immune response

## Abstract

This commentary wishes to highlight the 2018 Nobel Prize in Medicine awarded to two cancer immunotherapy scientists, Prof James Allison and Prof Tasuku Honjo, for their discovery on unleashing the body’s immune system to attack cancer. Their studies have led to the development of an entire class of drugs that hopefully will bring lasting remissions to many patients who had run out of options.

## Background

The Nobel Prize in medicine for 2018 was awarded to Prof James Allison of MD Anderson Cancer Center, USA, and Prof Tasuku Honjo of Kyoto University, Japan, for their discovery of cancer therapy by inhibition of negative immune regulation. Previously in 2014, they both received the first Tang Prize for biopharmaceutical science for their work, Prof Allison won the Lasker prize in 2015 and Prof Honjo won the Kyoto prize in basic sciences in 2016.

**Fig. 1:**
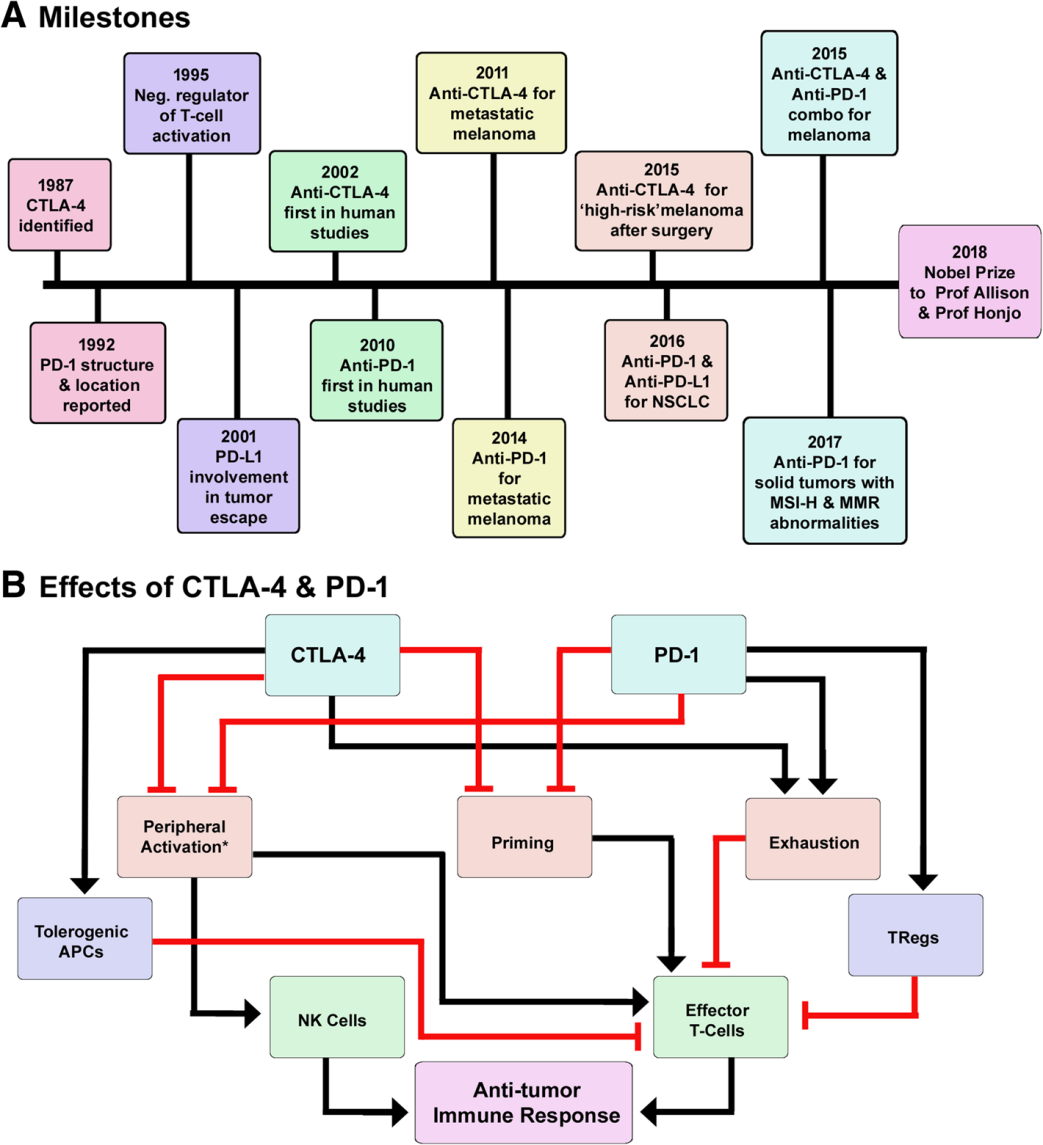
Milestones in cancer immunotherapy. **a** Schematic representation of the milestones in cancer immunotherapy in the last 20 years. **b** Schematic representation of the effects of CTLA-4 and PD-1 blockade. There is an overlap between the mechanisms by which CTLA-4 and PD-1 blockade stimulates immune response. Both the pathways are involved in T-cell priming, activation and exhaustion. PD-1 is involved in priming of TRegs whereas CTLA-4 induces tolerogenic phenotype in DCs. *NKcells do not express CTLA-4 and are only activated by PD-1 blockade

Immunologists have been trying to identify methods to activate immune system and drive anti-tumor immune response since long time. Prof Allison and Prof Honjo’s research helped in development of successful strategies to activate immune system and made tumor immunology a flourishing area of research. The Milestones in cancer immunotherapy are shown in Fig. 1a. Prof Allison is known for his work on cytotoxic T-lymphocyte-associated protein 4 (CTLA-4) also known as cluster of differentiation 152 (CD152), a receptor expressed mainly on activated lymphocytes. CTLA-4 was first discovered in 1987 as a protein belonging to immunoglobulin superfamily of proteins [1]. Its structure is strikingly similar to T-cell activating receptor, CD28. Both CTLA-4 and CD28 bind to same ligands, CD80 and CD86. Interestingly, CTLA-4 was initially thought to be a positive regulator of T-cells and to co-operate with CD28 in the activation of T-cells. Prof Allison’s research helped in clearly demonstrating the negative regulatory role of CTLA-4 and the opposing effects of CTLA-4 and CD28 in response to T-cell stimulation [2]. His lab showed that CTLA-4 engagement resulted in inhibition of IL-2 accumulation and cell cycle progression in activated T-cells and further confirmed the inhibitory role of CTLA-4 by illustrating lymphoproliferative and lethal autoimmune phenotype in Ctla-4−/− mice (Fig. 1b). More importantly, his work also demonstrated the potential of blocking CTLA-4 in the treatment of cancer [3].

Prof Honjo is well-known for the discovery of Programmed cell death protein 1, also known as PD-1 and CD279 (cluster of differentiation 279) and for elucidation of its functions. PD-1 gene was isolated using subtractive hybridization technique, while working on pathways of programmed cell death [4]. PD-1 is a cell surface receptor belonging to the immunoglobulin super family proteins that is expressed on T cells, B cells and natural killer (NK) cells. Prof Honjo worked extensively on PD-1 and demonstrated the immune inhibitory role of PD-1. His lab showed that lack of PD-1 results in comparatively milder autoimmune phenotype in mice that was dependent on the genetic background of the mice. He also collaborated with researchers across the world and contributed to the identification of ligands for PD-1 and showed the involvement of PD-1 ligands on tumor cells in escape from immune response [5, 6] (Fig. 1b).

In the past decade CTLA-4 and PD-1 have been found to be very reliable targets for the modulation of immune response and for the treatment of cancer. CTLA-4 and PD-1 blockade was shown to stimulate immune response via T-cell priming, peripheral activation of immune cells, reinvigoration of exhausted immune cells and inhibition of immunosuppressor cells such as regulatory T cells (TRegs) (Fig. 1b). Drugs targeting CTLA-4 and PD-1, commonly known as immune checkpoint blockers dramatically changed the treatment landscape for advanced cancers. Prior to the approval of anti-CTLA-4 monoclonal antibody, ipilimumab, metastatic melanoma patients had limited treatment options that had durable response rates and had poor prognosis with 5-year survival rate of less than 20% [7]. Long-term survival rates seen in ipilimumab-treated patients encouraged development of anti-PD-1 antibodies, nivolumab and pembrolizumab. Since their approval immune checkpoint blockers have extended the survival of melanoma patients by years and wiped out all signs of disease in some patients. One among such patients is President Jimmy Carter, who had remarkable recovery after being diagnosed with Stage IV melanoma that was metastasized to brain.

Apart from metastatic melanoma, anti-PD-1 antibodies are approved as ‘first-line’ therapy for advanced non small cell lung cancer, chronic Hodgkin’s lymphoma, head and neck squamous cell carcinoma, gastric cancer, urothelial cancer, cervical cancer, renal cell carcinoma and hepatocellular carcinoma [8]. They are also broadly approved for any solid tumor with microsatellite instability-high and mismatch repair deficiency. In addition to monotherapy, combination of CTLA-4 and PD-1 targeting antibodies has also been approved for metastatic melanoma and other types of cancers. Most importantly, the adverse events seen with immune checkpoint blockers are milder and manageable compared to the ones seen with conventional cancer treatments such as chemotherapy. Adverse events seen with immune checkpoint blockers are also reversed upon cessation of the therapy [9].

The significance of targeting PD-1 and other immune checkpoints for treatment of cancer can be seen by the interest from various pharmaceutical and biotech companies around the world. Almost every pharmaceutical R&D has immunotherapy in their pipeline with at least one immune checkpoint blocker under development. More than 30 monoclonal antibodies targeting PD-1 or its ligand PD-L1 are in advanced stages of development. The success of immune checkpoint blockers also paved the way for other types of immunotherapy such as chimeric antigen receptor engineered T-cells (CAR-T cells) and neoantigen based cancer vaccines which were previously considered as ‘high-risk’ projects for drug developers [10]. Three CAR-T cell based therapies and one oncolytic virus based therapy are approved for treatment of cancer and multiple new approaches are in clinical trials. Hundreds of new clinical trials have been initiated in the past 5 years to test new immune checkpoint blockers, new immunotherapeutic approaches as well as combinations of approved PD-1 blockers. The success of CTLA-4 and PD-1 blockade for cancer treatment has had a huge impact on the fields of oncology as well as immunology and the Nobel prize for Prof Allison and Prof Honjo is well deserved. It can be considered as recognition for the entire field of tumor immunology, which made surviving advanced stages of cancer ‘achievable’.

## Conclusion

Taken together, these discoveries hold great promises for those patients who had run out of options and, in this regard, *Journal of Experimental &Clinical Cancer Research* is announcing, for the end of the year, a special issue in Advances in Cancer Immunotherapy to collect knowledge and limitations of cancer immunotherapy.

## Abbreviations

CAR-T cells: Chimeric antigen receptor engineered T-cells
CD152: Cluster of differentiation 152
CD279: Cluster of differentiation 279
CTLA-4: Cytotoxic T-lymphocyte-associated protein 4
PD-1: Programmed cell death protein 1
TRegs: Regulatory T cells
